# Cactus-like architecture for synergistic microwave absorption and thermal management

**DOI:** 10.1093/nsr/nwaf394

**Published:** 2025-09-17

**Authors:** Jiamin Qi, Chaobo Liang, Kunpeng Ruan, Mukun Li, Hua Guo, Mukun He, Hua Qiu, Yongqiang Guo, Junwei Gu

**Affiliations:** Shaanxi Key Laboratory of Macromolecular Science and Technology, School of Chemistry and Chemical Engineering, Northwestern Polytechnical University, Xi’an 710072, China; Shaanxi Key Laboratory of Macromolecular Science and Technology, School of Chemistry and Chemical Engineering, Northwestern Polytechnical University, Xi’an 710072, China; Key Laboratory of Functional Nanocomposites of Shanxi Province, College of Materials, Science and Engineering, North University of China, Taiyuan 030051, China; Shaanxi Key Laboratory of Macromolecular Science and Technology, School of Chemistry and Chemical Engineering, Northwestern Polytechnical University, Xi’an 710072, China; Shaanxi Key Laboratory of Macromolecular Science and Technology, School of Chemistry and Chemical Engineering, Northwestern Polytechnical University, Xi’an 710072, China; Shaanxi Key Laboratory of Macromolecular Science and Technology, School of Chemistry and Chemical Engineering, Northwestern Polytechnical University, Xi’an 710072, China; Shaanxi Key Laboratory of Macromolecular Science and Technology, School of Chemistry and Chemical Engineering, Northwestern Polytechnical University, Xi’an 710072, China; Shaanxi Key Laboratory of Macromolecular Science and Technology, School of Chemistry and Chemical Engineering, Northwestern Polytechnical University, Xi’an 710072, China; Shaanxi Key Laboratory of Macromolecular Science and Technology, School of Chemistry and Chemical Engineering, Northwestern Polytechnical University, Xi’an 710072, China; Shaanxi Key Laboratory of Macromolecular Science and Technology, School of Chemistry and Chemical Engineering, Northwestern Polytechnical University, Xi’an 710072, China

**Keywords:** cactus-like heterogeneous structure, multifunctional composites, integrated thermal–electromagnetic management, thermal conductivity, microwave absorption

## Abstract

As electronic devices evolve toward miniaturization, integration and diversification, developing composites with thermal management and broadband microwave absorption has become critical for addressing electromagnetic compatibility and heat-dissipation challenges. Inspired by the multilevel thorny structure of a cactus, this study proposes a biomimetic 3D network structure via a ‘direction-decoupling’ design to enhance thermal conductivity and microwave absorption. Boron nitride nanosheets (BNNS) form horizontal thermal pathways, while cobalt-catalysed nitrogen-doped carbon nanotube arrays (Co@NCNTs) are vertically grown in the interlayer for cactus-like heterostructure fillers. Finally, composites are obtained by combining the solid–solid phase-change polyethylene glycol matrix with the directional assembly process. At a mass fraction of 30 wt% for (Co@NCNTs)@BNNS, the composites exhibit the best microwave absorption and thermal conductivity at a thickness of 2.5 mm. The maximum effective absorption bandwidth reaches 6.72 GHz, with in-plane and through-plane thermal conductivity coefficients reaching 2.55 and 0.94 W·m^–1^·K^–1^, realizing simultaneous improvements in thermal conductivity and microwave-absorption performance. Moreover, density functional theory analysis confirms the interfacial bonding between Co@NCNTs and BNNS systems and verifies the advantages of a unique electronic structure for microwave absorption between Co- and nitrogen-doped carbon nanotubes. This study provides new strategies for integrated thermal–electromagnetic management materials in next-generation high-density electronics.

## Teaser

Inspired by the cactus, the composite enables dual functionality in thermal management and microwave absorption through architecturally aligned heterogeneous fillers.

## INTRODUCTION

With the wide application of 5G communication and high-density electronic components, low heat-dissipation efficiency and electromagnetic pollution have become dual challenges restricting the stability of electronic components. The development of bifunctional materials with efficient thermal management and broadband microwave-absorption performance has become a key requirement for the heat-dissipation and electromagnetic compatibility of electronic components [1–4].

Among many carbon-based materials, carbon nanotubes (CNTs) have become a research hotspot for microwave-absorption materials due to their high conductivity and large specific surface area. However, the high dielectric constant of CNTs easily causes impedance mismatch and a single loss mechanism, limiting their application in complex electromagnetic environments [5–8]. Optimizing impedance matching and introducing different loss mechanisms can further enhance broadband microwave-absorption performance by combining magnetic components such as Fe₃O₄ [9,10] and CoNi alloys [11–13]. Melvin *et al.* constructed barium titanate@CNTs composites through sol–gel techniques, resulting in a minimum reflection loss (*RL*_min_) of –37.2 dB and a maximum effective absorption bandwidth (*EAB*_max_) of 1.6 GHz at a thickness of 1.1 mm [14]. However, there are some problems, such as particle agglomeration, weak interface bonding and poor microwave-absorption performance when CNTs are compounded with conventional magnetic metals. In contrast, the combination of metal–organic framework (MOF) derivatives and CNTs can achieve precise control of the structure, showing better microwave-absorption performance [15–17]. Wang *et al.* prepared Co@C/CNTs composites through ZIF-67 derivatization, achieving an *EAB*_max_ of 5.4 GHz and *RL*_min_ of –57.6 dB at a thickness of 2 mm [18]. The introduction of MOF derivatives can effectively improve the microwave-absorption performance of composites. However, the porous interfaces and defect structure introduced by such composites will aggravate phonon scattering, resulting in a sharp decline in thermal conductivity. It is difficult to meet the collaborative needs of high-power devices such as 5G base stations, AI chips and radar systems for heat-dissipation and electromagnetic compatibility [19]. Therefore, it is of core significance to realize the synergistic enhancement of thermal conductivity and microwave-absorption performance to promote the engineering application of high-performance electronic equipment.

As a typical 2D material, boron nitride nanosheets (BNNS) have shown significant advantages in thermal conductivity and microwave absorption [20,21]. In terms of thermal conductivity, BNNS have excellent anisotropic thermal conductivity, with an in-plane thermal conductivity (*λ_∥_*) of ≤400 W·m^–1^·K^–1^ and a through-plane thermal conductivity (*λ_⊥_*) of ~30 W·m^–1^·K^–1^. In terms of microwave absorption, the low dielectric properties of BNNS can alleviate the impedance mismatch problem of carbon-based materials. The large specific surface area can enhance the interfacial polarization effect, further improving the microwave-absorption performance of composites [22]. Mou *et al.* used the ‘ball-milling–high-temperature pyrolysis’ method to prepare biomass-derived boron nitride (BCN) nanosheets, which were blended with natural rubber (NR) to prepare BCN/NR composites [23]. When the thickness of the composites was 1.4 mm, the *EAB*_max_ and *RL*_min_ were 4.16 GHz and –54.2 dB, respectively, and the *λ_⊥_* was 0.338 W·m^–1^·K^–1^. It is evident that the extensive interface facilitates the enhancement of microwave polarization loss at the composites interface, improving microwave-absorption performance. Nevertheless, the interface also induces phonon boundary scattering, leading to an increase in thermal resistance and consequently impeding the effective transmission of phonons. In order to enhance the thermal conductivity of composites, it is essential to establish continuous and intact thermal conduction pathways while minimizing structural defects [24–27]. Therefore, a microstructure design is expected to provide a solution for both thermal conductivity and microwave absorption [28–31].

The cactus has successfully adapted to the harsh temperature fluctuations and high-intensity radiation of the desert environment thanks to its unique multilevel structure. The horizontal folded epidermis reduces heat absorption through a slow reflection mechanism, cone-shaped spines disturb airflow to enhance heat dissipation and the tube bundle network inside the stem simultaneously transports water and provides mechanical support. Inspired by cactus structures, a multilevel structure offers great potential in the fields of thermal management and microwave absorption. The horizontal lamellar fold structure constructs a continuous thermal conduction channel in the horizontal direction. At the same time, the microwave-attenuation pathways are broadened by multiple interfacial reflections. The vertically oriented conical array establishes an axial heat-dissipation channel. It uses the vertical direction to build a 3D continuous conductive network to realize the multiple losses of microwaves so as to achieve the unity of thermal conductivity and microwave-absorption performance.

Solid–solid phase-change materials can achieve controlled heat release through reversible changes in molecular chain conformation, thereby alleviating the thermal shock caused by high heat flux on thermally conductive materials [32,33]. Based on this, a solid–solid phase-change polyethylene glycol (ScPEG) matrix is first designed to store and release heat through reversible changes in the microstructure, effectively delaying sudden temperature rises and enabling dynamic thermal management. Subsequently, cobalt-catalysed nitrogen-doped carbon nanotubes (Co@NCNTs) are combined with BNNS by using the ‘*in situ* growth–thermal reduction’ method to prepare cactus-like (Co@NCNTs)@BNNS heterostructure fillers. Finally, the (Co@NCNTs)@BNNS/ScPEG composites are constructed based on the directional assembly techniques of ‘bidirectional freezing–vertical hot pressing’. Specifically, BNNS are used as a directional thermal conduction skeleton to construct a rapid heat-conduction channel that is similar to a cactus stem. By simulating the directional regulation mechanism of airflow on the multilevel spiny surface of the cactus, Co@NCNTs are grown *in situ* between BNNS layers, utilizing multiple loss mechanisms to achieve efficient microwave absorption. X-ray diffraction (XRD), scanning electron microscopy (SEM) and transmission electron microscopy (TEM) are used to analyse and characterize the structures and morphologies of ZIF-67, ZIF-67@BNNS and (Co@NCNTs)@BNNS. The effects of the mass ratio of BNNS to Co^2+^ and the amount of (Co@NCNTs)@BNNS on the microwave-absorption performance of composites are analysed. At the same time, the effects of the orientation structure of (Co@NCNTs)@BNNS and the Co@NCNTs array on the thermal conductivity of composites are analysed. In addition, a density functional theory (DFT) analysis proves the interfacial bonding of the Co@NCNTs and BNNS systems. It also verifies the advantages of the unique electronic structure between the Co and NCNTs for microwave absorption.

## RESULTS AND DISCUSSION

### Structure and morphologies of (Co@NCNTs)@BNNS fillers

**Figure 1.**
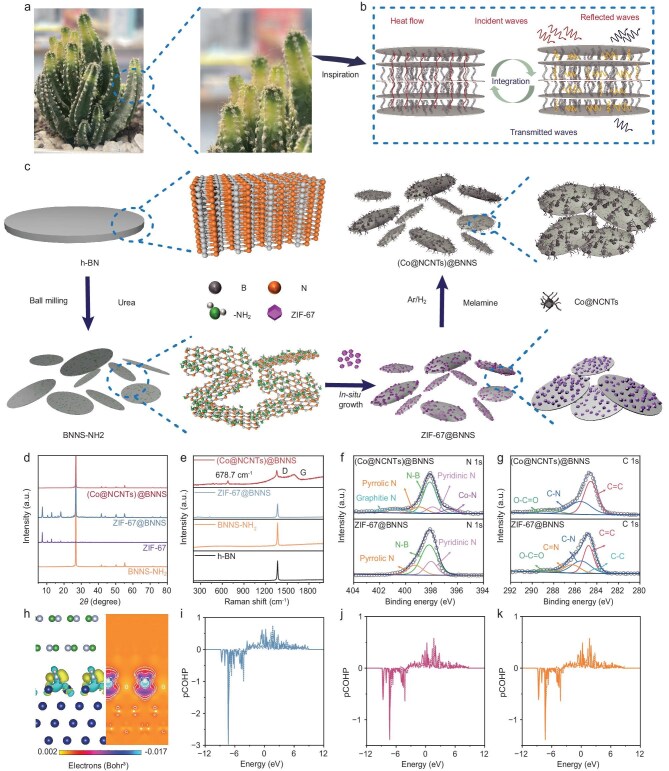
(a) Cactus images and (b) structure inspiration. (c) Schematic diagram of cactus-like (Co@NCNTs)@BNNS. (d) XRD of BNNS–NH_2_, ZIF-67, ZIF-67@BNNS and (Co@NCNTs)@BNNS. (e) Raman of h-BN, BNNS–NH_2_, ZIF-67@BNNS and (Co@NCNTs)@BNNS. (f) N 1s spectra and (g) C 1s X-ray photoelectron spectroscopy (XPS) spectra of ZIF-67@BNNS and (Co@NCNTs)@BNNS. (h) Charge-density diagram for Co-BNNS. (i–k) Projected crystal orbital Hamilton population (pCOHP) curves.

Inspired by the multilevel structure of a cactus, the heterostructure fillers are designed and fabricated (Fig. 1a and b). Firstly, amino-grafted BNNS (BNNS–NH₂) are prepared through ball milling. Subsequently, ZIF-67 is grown *in situ* on the surface of BNNS–NH_2_ at room temperature. Then, Co^2+^ is reduced to Co nanoparticles by using the thermal reduction method. Finally, Co atoms act as a catalyst, utilizing the nitrogen and carbon sources provided by melamine to facilitate the *in situ* growth of Co@NCNTs on the BNNS surface, resulting in cactus-like (Co@NCNTs)@BNNS heterostructure fillers (Fig. 1c).

Figure S1 illustrates the fourier transform infrared spectra (FTIR) of h-BN and BNNS–NH₂. The peaks at 1277 and 750 cm^−^¹ correspond to B–N and B–N–B stretching peaks. Compared with h-BN, the ball-milled BNNS show a broad peak at ~3400 cm^−^¹, which confirms the N–H stretching peak [34]. This indicates the presence of amino groups on the surface of the BNNS after ball milling. *In situ* binding of ZIF-67 to BNNS–NH_2_ (ZIF-67@BNNS) is the primary process for the preparation of cactus-like heterostructure fillers. As shown in Fig. 1d, the XRD spectra of ZIF-67@BNNS include all the characteristic peaks of ZIF-67 and BNNS–NH_2_. Also, the peak position of ZIF-67@BNNS shows no shift compared with that of BNNS–NH_2_ and ZIF-67 alone. This is because BNNS and ZIF-67 bind through weak interaction, which does not affect the crystal structures of BNNS–NH_2_ and ZIF-67. Raman spectra of (Co@NCNTs)@BNNS (Fig. 1e) exhibit characteristic D (1350 cm^−^¹) and G (1600 cm^−^¹) peaks, confirming the formation of graphitized NCNTs through Co-catalysed growth. Figure S2a shows a red-shifted B–N peak (1365 cm^−^¹) in (Co@NCNTs)@BNNS, attributed to the increased interlayer spacing of BNNS induced by Co@NCNTs growth [35]. In addition, two new characteristic peaks of Co–N (396.6 eV) and graphite N (401.5 eV) appear (Fig. 1f), while the two characteristic peaks of C=N (286.2 eV) and C–C (284.0 eV) disappear in (Co@NCNTs)@BNNS (Fig. 1g). These results indicate the interaction between the Co and BNNS. This result is further confirmed by using Raman spectra, in which the peak at 678.7 cm^−1^ corresponds to the vibrational peak of the Co–N bond, indicating that Co atoms have successfully anchored to the BNNS surface to form a stable chemical bond (Fig. S2b). DFT calculations illustrate the interfacial bonding between Co and BNNS, and the optimized model is shown in Fig. S3 [36]. Figure 1h demonstrates significant electron density accumulation at the Co–N interface, where the atomic spacing of 1.94 Å is consistent with the bond-length characteristic of the chemical bond [37]. The crystal orbital Hamiltonian population (pCOHP) is used for further analysis of the Co–N bond. The pCOHP exhibits a low negative value (pCOHP < –1 eV), indicating the presence of interfacial bonding between the Co and BNNS (Fig. 1i–k) [38].

**Figure 2.**
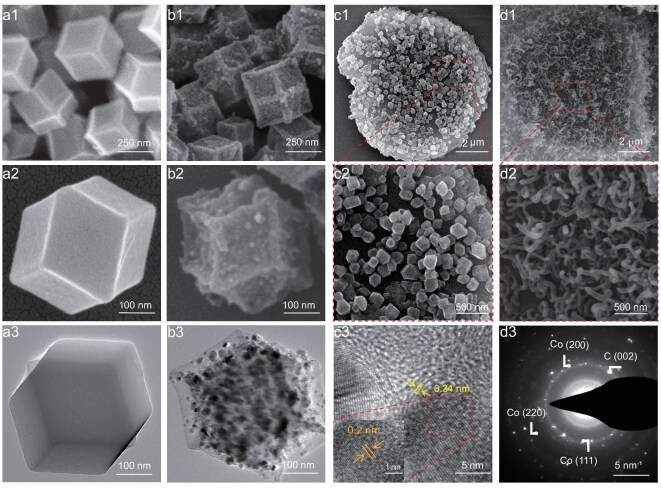
SEM images of (a1, a2) ZIF-67, (b1, b2) Co@C, (c1, c2) ZIF-67@BNNS and (d1, d2) (Co@NCNTs)@BNNS. TEM images of (a3) ZIF-67 and (b3) Co@C. (c3) High-resolution TEM image and (d3) SAED image of Co@NCNTs.

Figure 2 shows the SEM and TEM images of ZIF-67, ZIF-67@BNNS and their corresponding pyrolysis products. Figure 2a illustrates that the ZIF-67 has a consistent size, smooth surface and dodecahedral form, with particle dimensions of ~250 nm. Although ZIF-67 shrinks to a certain extent in the pyrolysis product (Co@C), it can still maintain the shape and size of the dodecahedron (Fig. 2b). As shown in Fig. S4, the diameter of BNNS after ball milling is ~15 µm and a large aspect ratio is conducive to the construction of a line-surface structure. The surface of the BNNS is uniformly covered with a ZIF-67 nanocrystalline layer and the crystal size is uniform and evenly distributed, as shown in Fig. 2c1 and c2. In addition, the element mapping shown in Fig. S5 further demonstrates the successful uniform growth of ZIF-67 nanocrystals on the BNNS surface. High-temperature pyrolysis reduces Co^2+^ to highly active Co nanocatalysts that capture carbon and nitrogen atoms to grow NCNTs *in situ* on the surface of the BNNS. As shown in Fig. 2d1 and d2, a large number of NCNTs are uniformly grown on the surface of the BNNS, forming cactus-like (Co@NCNTs)@BNNS heterostructure fillers. Furthermore, the tips of the NCNTs are coated with Co nanoparticles that are based on the tip-growth mechanism (Fig. S6). High-resolution TEM (Fig. 2c3) reveals lattice spacings of 0.20 and 0.34 nm, corresponding to Co (111) and graphitic C (002) planes, respectively. It confirms the graphitic carbon coated around Co nanoparticles [39]. Figure S7 further confirms through TEM and energy dispersive X-ray spectroscopy (EDS) that Co nanoparticles are encapsulated within NCNTs. The average length of the NCNTs catalysed on the BNNS surface was ~500 nm. Simultaneously, selected area electron diffraction (SAED) patterns show two weak diffraction rings belonging to the Co (200) and (220) crystal planes, which is consistent with the XRD results (Fig. 2d3) [18].

**Figure 3.**
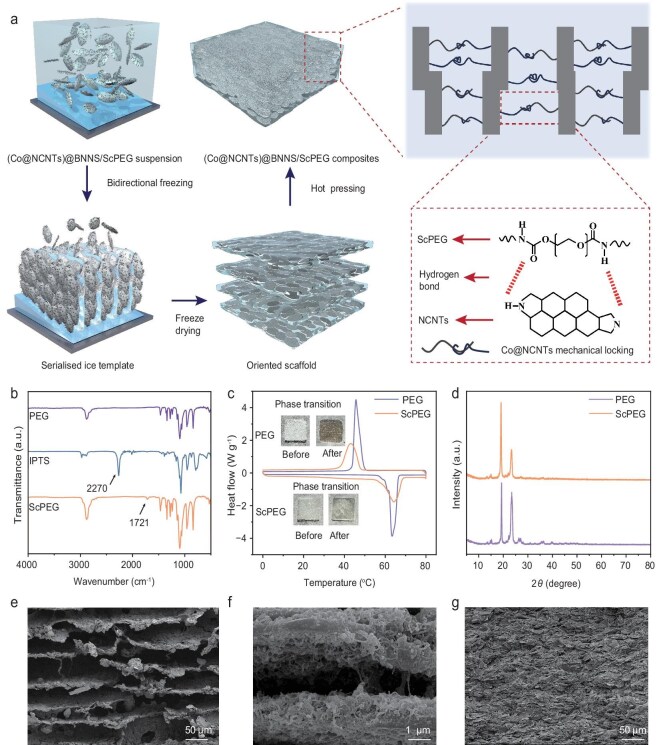
(a) Schematic illustration of the fabrication process of the (Co@NCNTs)@BNNS/ScPEG composites. (b) FTIR spectra of PEG, IPTS and ScPEG. (c) DSC curves of PEG and ScPEG. (d) XRD of PEG and ScPEG. Cross-sectional SEM images for (e, f) (Co@NCNTs)@BNNS/ScPEG skeleton and (g) composites.

Figure 3a illustrates the fabrication process of the (Co@NCNTs)@BNNS/ScPEG composites. First, highly reactive silanol groups are introduced into the PEG terminals, which are then condensed to obtain a shape-stable solid–solid phase-change matrix (Fig. S8). Specifically, the PEG molecular chain functions as a functional flexible segment to store and release heat energy by converting crystalline and amorphous states. The silanol group functions as a connecting hard segment to form a supramolecular network through the physical cross-linking of multiple hydrogen bonds, resulting in good mechanical strength and thermal stability (Fig. S9). Subsequently, (Co@NCNTs)@BNNS/ScPEG composites are prepared based on bidirectional freezing and hot-pressing techniques.

Figure 3b shows the FTIR of polyethylene glycol (PEG), 3-isocyanatopropyltriethoxysilane (IPTS) and ScPEG. In the ScPEG spectra, the N=C=O stretching peak at 2270 cm^−1^ completely disappears and the C=O stretching peak at 1721 cm^−1^ appears, indicating that the hydroxyl group of PEG has successfully reacted with the isocyanate group of IPTS [18,40]. Figure 3c illustrates the differential scanning calorimetry (DSC) curves for PEG and ScPEG. The melting enthalpy (Δ*H*_m_) and crystallization enthalpy (Δ*H*_c_) of PEG are 162.7 and 161.1 J/g, respectively. In contrast, the Δ*H*_m_ and Δ*H*_c_ of ScPEG decrease to 127.8 and 123.3 J/g, respectively. This is because the Si–O–Si cross-linking network hinders the thermal motion of the PEG chain. Although the enthalpy value of ScPEG is lower than that of PEG, its thermal stability is greatly improved. As shown in the inset of Fig. 3c, when the temperature exceeds the phase-change point of PEG and ScPEG, both transition from opaque to transparent, indicating a change from a crystalline state to an amorphous state. ScPEG maintains the original solid state, while PEG changes into a liquid state. Figure 3d shows the XRD of PEG and ScPEG. Both exhibit characteristic peaks at 19.1° and 23.2°, corresponding to the (120) and (130) crystal planes of PEG triclinic crystals, which indicates that ScPEG inherits the crystalline properties of PEG [41]. Additionally, compared with PEG, ScPEG has Si element characteristic peaks (Fig. S10a). The presence of Si–O–Si (102.3 eV), Si–C (101.6 eV) and Si–OH (103 eV) peaks further indicates the successful synthesis of ScPEG (Fig. S10b) [18]. Figure 3e and f shows the SEM images of the oriented (Co@NCNTs)@BNNS/ScPEG framework. The BNNS are arranged horizontally and Co@NCNTs overlap between the BNNS layers to form a 3D network based on a ‘line-surface’ structure. The oriented framework formed through hot pressing has a dense and highly ordered structure, which provides a structural basis for the orderly construction of heat-conduction pathways and multiple losses of microwaves in composites (Fig. 3g).

### Microwave-absorption performance of (Co@NCNTs)@BNNS/ScPEG composites

**Figure 4.**
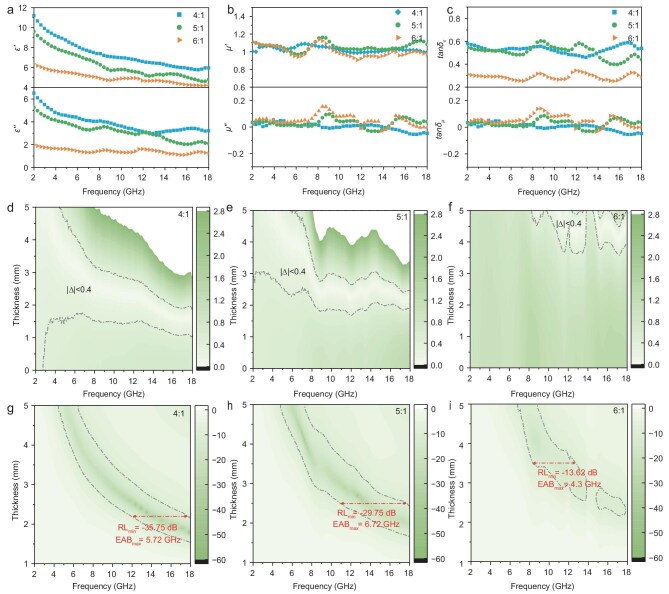
(a) *ε′* and *ε″*, (b) *μ′* and *μ″*, and (c) tan*δ_ε_* and tan*δ_μ_* of (Co@NCNTs)@BNNS/ScPEG composites. (d–f) Impedance matching of (Co@NCNTs)@BNNS/ScPEG composites. (g–i) *RL*_min_ and *EAB*_max_ of (Co@NCNTs)@BNNS/ScPEG composites.

Figure S11 compares the *EAB*_max_ of (Co@NCNTs)@BNNS/ScPEG composites with mass fractions ranging from 10 to 50 wt%. It can be seen that, for (Co@NCNTs)@BNNS at a dosage of 30 wt%, the composites have the optimal *EAB*_max_. Therefore, in this work, the microwave-absorption performance of the composites is optimized based on a (Co@NCNTs)@BNNS dosage of 30 wt%. Figure 4 shows the real part (*ε*′) and imaginary part (*ε*″), complex permeability real part (*μ*′) and imaginary part (*μ*″), *RL* and *EAB* of the (Co@NCNTs)@BNNS/ScPEG composites when the mass ratios of BNNS to Co^2+^ are 4:1, 5:1 and 6:1. As shown in Fig. 4a–c, when the mass ratio of BNNS to Co^2+^ increases from 4:1 to 6:1, the *ε*′, *ε*″ and dielectric loss (tan*δ_ε_*) of the composites decrease, while the *μ*’, *μ*″ and magnetic loss (tan*δ_μ_*) fluctuate very little. This is due to the relatively low content of Co metal particles in (Co@NCNTs)@BNNS and the small difference in magnetic responsiveness (Fig. S12). Additionally, the tan*δ_ε_* of the three composites is significantly larger than that of tan*δ_μ_*, suggesting that the primary mechanism of microwave absorption is dielectric loss (Fig. 4c).

Figure S13 shows the attenuation constants for three composites (*α*, Equation (S4)). When the mass ratios of BNNS and Co^2+^ are 4:1 and 5:1, the *α* values of the composites are similar because the increase in BNNS content does not destroy the ‘line-surface’ structure and conductive network of the heterostructure fillers, so the dissipation capacity of microwaves is only slightly different. When the mass ratio of BNNS to Co^2+^ reaches 6:1, the *α* value of the composites decreases significantly. This is because the impedance matching between the composites and free space is poor and microwaves cannot be sufficiently attenuated. Figure 4d and f shows the impedance-matching parameters (|Δ|, Equation (S3)) for three composites, where |Δ|<0.4 represents a good impedance match [42]. It can be observed that the range of the optimal impedance-matching region decreases with an increase in the BNNS content. The microwave-absorption performances of three composites are calculated by using Equations (S1) and (S2), as illustrated in Fig. 4g–i. When the mass ratios of BNNS to Co^2+^ are 4:1 and 6:1, the *RL*_min_ of the composites are –35.75 and –13.62 dB, with corresponding *EAB*_max_ values of 5.72 and 4.3 GHz. Notably, at a mass ratio of BNNS to Co^2+^ of 5:1, the composites exhibit optimal microwave-absorption performance. The *RL*_min_ and *EAB*_max_ are –29.75 dB and 6.72 GHz, respectively, covering the entire Ku-band and part of the X-band. The above results show that the microwave-attenuation capability and impedance-matching characteristics of (Co@NCNTs)@BNNS/ScPEG composites can be effectively adjusted to achieve the best microwave-absorption performance by controlling the mass ratio of BNNS to Co^2+^.

**Figure 5.**
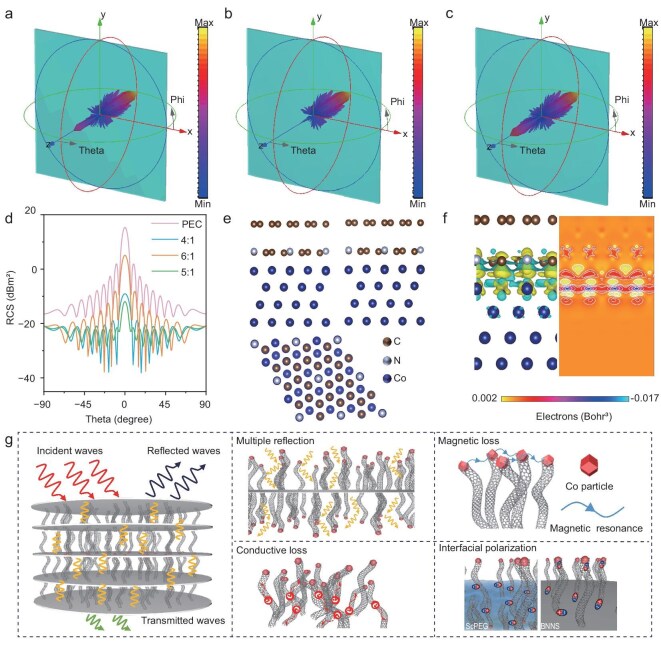
(a–c) 3D RCS plots of the PEC plane covered with (Co@NCNTs)@BNNS/ScPEG composites prepared when the mass ratios of BNNS to Co^2+^ were 4:1, 5:1 and 6:1. (d) Simulated RCS curves of the PEC plane and the PEC plane covered with (Co@NCNTs)@BNNS/ScPEG composites with varying incidence angles. (e) Optimized structural model of Co@NCNTs. (f) Charge-density diagram for Co@NCNTs. (g) Schematic illustration of microwave-absorption mechanism of (Co@NCNTs)@BNNS/ScPEG composites.

Figure 5a–c presents a 3D diagram of the radar scattering cross section (RCS) simulation of the (Co@NCNTs)@BNNS/ScPEG composites, which characterizes the actual far-field absorption capacity of the materials. The scattered signal is shown as a blue bar graph, indicating the intensity of the electromagnetic waves emitted by the source after passing through the material. The more blue the color, the smaller the area of the graph, indicating that the material has a stronger ability to absorb electromagnetic waves. It is evident that, when the mass ratio of BNNS to Co^2+^ is 5:1, the composites exhibit the lowest scattering signal, demonstrating the best microwave-absorption capability [43,44]. This observation is further confirmed by 2D curves simulating the RCS in the Y–O–Z plane (Fig. 5d). Moreover, the RCS values obtained by subtracting the perfect electric conductor (PEC) plate also prove that the composites with a mass ratio of BNNS to Co^2+^ of 5:1 have the optimal microwave-absorption capacity (Fig. S14). In order to investigate the existence of charge transfer between Co and NCNTs, DFT was used to theoretically calculate the Co@NCNTs. As shown in Fig. 5e, the computational model is first established and optimized based on the microscopic characterization structure [45].

The differential charge-density plot for the Co@NCNTs is shown in Fig. 5f, with the yellow area representing the charge accumulation and the green area representing the charge consumption. There is a large yellow area on the surface of the graphitic carbon layer of the NCNTs, while the green area near the surface of the metal Co comprises the majority. These results indicate that electrons are transferred from the metal Co to the adjacent graphitic carbon layer, leading to a positive charge on the surface of the metal Co and a negative charge on the surface of the graphitic carbon layer of the NCNTs [46]. Therefore, there is interface polarization between the Co and NCNTs, which generates polarization loss under the action of an alternating electric field, thereby improving the microwave-absorption performance of the composites.

To further explore the microwave-absorption mechanism of (Co@NCNTs)@BNNS/ScPEG composites in a frequency range of 2~18 GHz, the contribution of dielectric loss and magnetic loss to microwave absorption is analysed. As shown in Fig. S15, the *ε*′–*ε*″ curves of the composites all have multiple relaxation peaks, indicating that, in addition to the conductive losses caused by Co@NCNTs, polarization losses are also an important mechanism for the composites to dissipate microwaves. In order to distinguish the contribution of conductive losses from polarization losses, Cole–Cole circles are drawn according to the Debye relaxation theory [47–49]. Each semicircle of the curve represents a Debye relaxation process, while the linear part reflects the conductive losses. When the mass ratio of BNNS to Co^2+^ is 5:1, the spectral lines of the composites near 17.0, 15.0, 12.0 and 9.0 GHz represent multiple polarization relaxation processes, corresponding to the relaxation peaks in the *ε*′–*ε*″ curves. This is mainly due to vacancy defects in the Co@NCNTs lattice and dipole polarization formed by the doping of nitrogen atoms. At the same time, Co@NCNTs introduce rich heterointerface structures on the BNNS surface, thereby synergistically enhancing the microwave-loss capability of composites. As shown in Fig. 4b, in terms of the magnetic loss mechanism, the *μ*″ curve shows resonance peaks in the high-frequency band (8–18 GHz), indicating the existence of natural resonance and exchange resonance [50,51]. The C_0_ values of the composites calculated by using Equation (S5) fluctuate over the whole test frequency range, indicating that the eddy current loss contributes very little to the magnetic loss. This further suggests that magnetic losses in this system mainly originate from natural resonance and exchange resonance mechanisms (Fig. S16). Based on this, the absorption mechanism of composites is briefly analysed, as shown in Fig. 5g. Firstly, microwaves enter the interior of the (Co@NCNTs)@BNNS/ScPEG composites. The orientation structure at the microscopic scale causes multiple reflections of microwaves, prolonging the loss pathways and thereby enhancing the loss capability. Secondly, there are multiple interfaces between the heterostructure fillers themselves and the matrix, which enhance the interfacial polarization loss capacity. Finally, the Co@NCNTs grown *in situ* on the BNNS surface provide abundant channels for electron transport and enhance the conductive loss capacity.

### Thermal management properties of (Co@NCNTs)@BNNS/ScPEG composites

**Figure 6.**
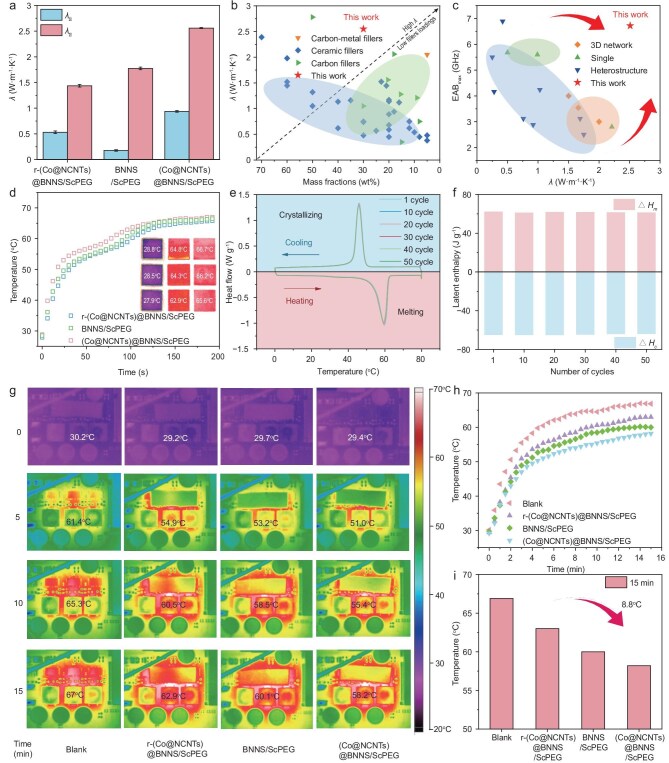
(a) *λ_⊥_* and *λ_∥_* of (Co@NCNTs)@BNNS/ScPEG, BNNS/ScPEG and r-(Co@NCNTs)@BNNS/ScPEG composites. (b) Comparison of *λ* with already-reported materials. (c) Comparison of *λ* and *EAB*_max_ with already-reported materials. (d) Infrared images of composites under heating conditions and the corresponding temperature profiles. (e) DSC curves from 1–50 heating–cooling cycles of (Co@NCNTs)@BNNS/ScPEG composites. (f) Enthalpy of the (Co@NCNTs)@BNNS/ScPEG composites during 1–50 continuous heating–cooling cycles. (g) Infrared images and (h) corresponding temperature profiles of motherboards made by using three types of composites during the overclocking operation stage. (i) Surface temperatures of the overclocking computer motherboards after 15 min of operation.

In order to explore the effect of the orientation structure and Co@NCNTs on the thermal conductivity of (Co@NCNTs)@BNNS/ScPEG composites, r-(Co@NCNTs)@BNNS/ScPEG composites (prepared by blending) and BNNS/ScPEG composites are used as control groups for comparison. Figure 6a shows that the *λ_∥_* and *λ_⊥_* of the (Co@NCNTs)@BNNS/ScPEG composites are 2.55 and 0.94 W·m^−1^·K^−1^, respectively, which are significantly higher than those of the r-(Co@NCNTs)@BNNS/ScPEG and BNNS/ScPEG composites. This is because BNNS are continuously stacked in the horizontal direction to form low thermal resistance pathways, while Co@NCNTs are oriented and overlapped in the vertical direction to form continuous heat-conduction pathways. It is noteworthy that the *λ_⊥_* of the BNNS/ScPEG composites (0.18 W·m^−1^·K^−1^) is lower than that of the r-(Co@NCNTs)@BNNS/ScPEG composites (0.52 W·m^−1^·K^−1^), attributed to the parallel arrangement of BNNS in the BNNS/ScPEG composites. The vertical thermal conductivity depends on the weak coupling of van der Waals forces between the layers, resulting in strong scattering of phonons between the layers and a significant decrease in thermal conductivity. Although r-(Co@NCNTs)@BNNS/ScPEG composites are randomly oriented, some (Co@NCNTs)@BNNS easily overlap to form local thermal conductivity networks [35,18]. Figure 6b and Table S1 present a *λ* comparison of different types of composites. The (Co@NCNTs)@BNNS/ScPEG composites demonstrate exceptional thermal conductivity at a low filler amount of 30 wt%. Simultaneously, as a type of integrated polymer composite with thermal conductivity and microwave-absorption functions, its performance evaluation needs to comprehensively consider the two key indicators. As illustrated in Fig. 6c and Table S2, the (Co@NCNTs)@BNNS/ScPEG composites exhibit an *EAB*_max_ of 6.72 GHz and a *λ_∥_* of 2.55 W·m^−1^·K^−1^, which are superior to those of most previously reported materials with dual functionalities. The temperature variations of the three composites are observed by using infrared cameras to further investigate heat-conduction performance.

Figure 6d shows that the surface temperature of the (Co@NCNTs)@BNNS/ScPEG composites is consistently the highest throughout the heating process. The result demonstrates that the horizontal network configuration of BNNS and the vertical arrangement of Co@NCNTs promote efficient heat conduction. The composites show a significant temperature lag region at ~60°C, due to the storage of heat energy generated by the ScPEG matrix during the phase change. This indicates that the prepared composites can respond dynamically according to a change in the heat-source temperature, avoiding the performance degradation or functional failure of electronic components due to sudden heat during operation. Furthermore, the ScPEG matrix can reduce the hardness of composites by absorbing heat energy, thereby reducing the contact thermal resistance (Fig. S17). The DSC curves of the (Co@NCNTs)@BNNS/ScPEG composites are shown in Fig. 6e. During 50 heating and cooling cycles, the phase-change temperatures of the composites remain almost constant, and the Δ*H*_m_ and Δ*H*_c_ remain at around 65.1 and 62.4 J/g (Fig. 6f), respectively, demonstrating that the (Co@NCNTs)@BNNS/ScPEG composites have excellent thermal and shape stability (Fig. S18). The central processing unit is the core device of electronic devices and its high-power operation generates a lot of heat. If heat energy is not dissipated in time, frequency downscaling can be triggered within seconds, causing the device to freeze and significantly accelerating chip aging [52–54]. Therefore, efficient thermal management of electronic devices is essential. Three composites are applied to a computer motherboard and infrared cameras are used to monitor temperature changes during the overclocking phase (Fig. S19). As shown in Fig. 6g and h, the rise in the surface temperature of the computer motherboard is the slowest and the equilibrium temperature is the lowest when the (Co@NCNTs)@BNNS/ScPEG composite is used as the heat-sink component. Compared with the blank control group, the surface equilibrium temperature of the computer motherboard is reduced by 8.8°C after 15 min of operation when using the (Co@NCNTs)@BNNS/ScPEG composite as the heat-dissipation component (Fig. 6i). These results show that the (Co@NCNTs)@BNNS/ScPEG composites, as thermal interface materials between computer motherboards and heat sinks, can effectively reduce the heat accumulation inside electronic components and then significantly improve the heat-dissipation performance.

## CONCLUSION

In summary, a solid–solid phase-change ScPEG matrix based on multiple hydrogen bonds is proposed. The cactus-like (Co@NCNTs)@BNNS heterostructure fillers are prepared by using the ‘*in situ* growth–thermal reduction’ method. Also, the Co@NCNTs@BNNS/ScPEG composites are constructed by using the directional assembly process of ‘bidirectional freezing–vertical hot pressing’. The density of the *in situ* grown Co@NCNTs on the BNNS surface is adjusted by adjusting the mass ratio of BNNS to Co^2+^ and the thermal conductivity and microwave-absorption performance of the composites are synergistically optimized. The composites demonstrate the best thermal conductivity and microwave-absorption performance when the mass ratio of BNNS to Co^2+^ is 5:1 and the mass fraction of (Co@NCNTs)@BNNS is 30 wt%. The *EAB*_max_ of the composites is 6.72 GHz, covering the entire Ku-band and part of the X-band. Simultaneously, the *λ_∥_* and *λ_⊥_* of composites reach 2.55 and 0.94 W·m^−1^·K^−1^, both of which are superior to those of composites prepared by direct blending with the same mass fraction. Furthermore, the composites exhibit Δ*H*_m_ and Δ*H*_c_ values of 65.1 and 62.4 J/g, demonstrating excellent latent heat storage and thermal response capability, which can effectively mitigate the performance degradation or functional failure of electronic components caused by sudden temperature changes. Therefore, the (Co@NCNTs)@BNNS/ScPEG composites show significant prospects in high-power electronic components such as 5G base stations, AI chips and radar systems due to their excellent thermal conductivity and microwave-absorption performance.

## METHODS

Experimental methods are available in the online Supplementary Data.

## Supplementary Material

nwaf394_Supplemental_File
