# Supplementary material for: Cactus-like architecture for synergistic microwave absorption and thermal management
Source: Natl Sci Rev. 2025 Sep 17;12(11):nwaf394. doi: 10.1093/nsr/nwaf394 (PMC12613159; doi:10.1093/nsr/nwaf394)
Supplement: nwaf394_Supplemental_File [file nwaf394_supplemental_file.pdf]

Cactus-like architecture for synergistic microwave absorption and thermal management

Jiamin Qi[a], Chaobo Liang[a,b,*], Kunpeng Ruan[a], Mukun Li[a], Hua Guo[a], Mukun He[a], Hua Qiu[a], Yongqiang Guo[a] and Junwei Gu[a,*]

[a]Shaanxi Key Laboratory of Macromolecular Science and Technology, School of Chemistry and Chemical Engineering, Northwestern Polytechnical University, Xi'an 710072, China;

[b]Key Laboratory of Functional Nanocomposites of Shanxi Province, College of Materials, Science and Engineering, North University of China, Taiyuan 030051, China

[*]**Corresponding authors.** E-mails: gjw@nwpu.edu.cn; nwpugjw@163.com; lcb@nuc.edu.cn

Results and discussion

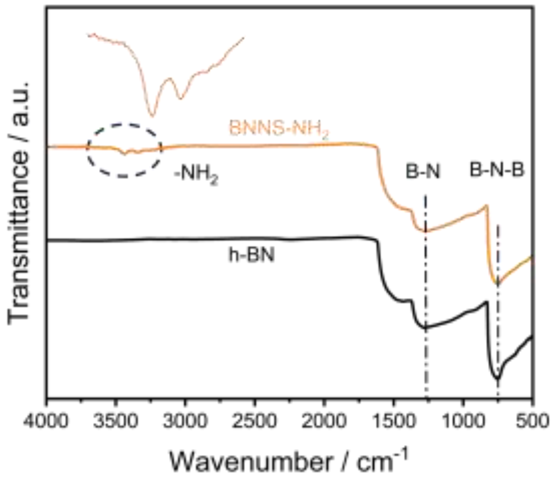

Figure S1 The FT-IR spectra of h-BN and BNNS-NH_2.

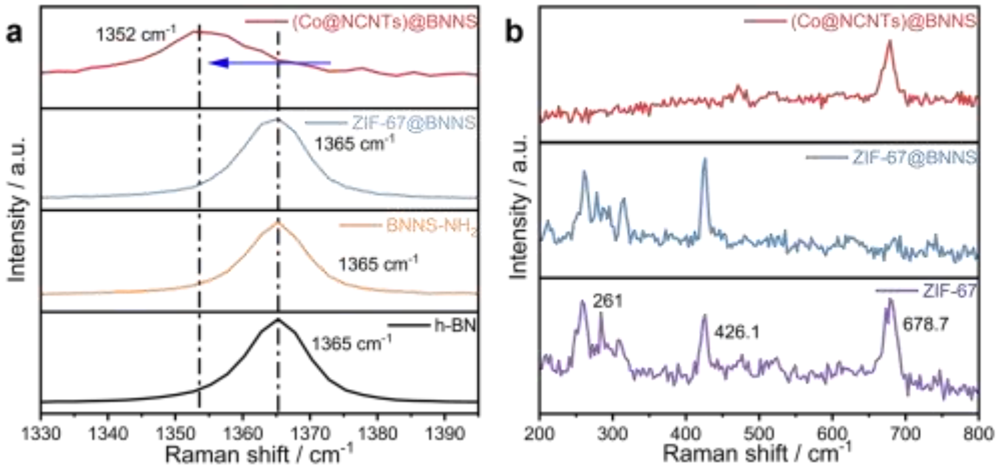

Figure S2 The Raman of h-BN, BNNS-NH_2, ZIF-67, ZIF-67@BNNS and (Co@NCNTs)@BNNS.

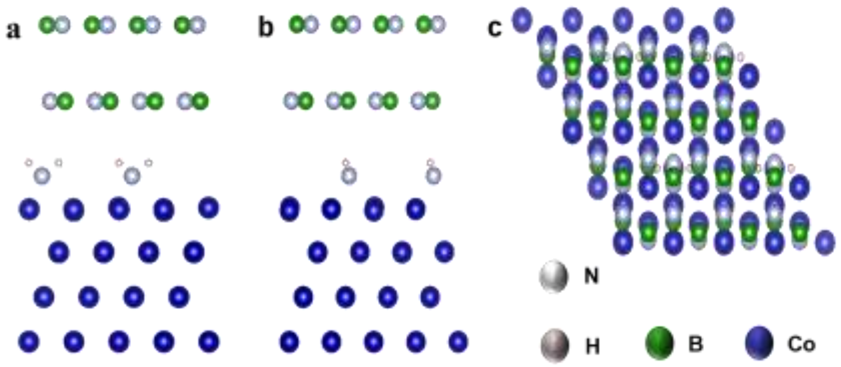

Figure S3 Main (a), left (b) and top (c) views of the optimized structure of Co-BNNS.

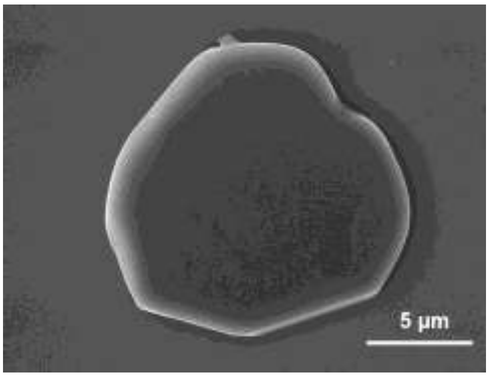

Figure S4 The SEM of BNNS.

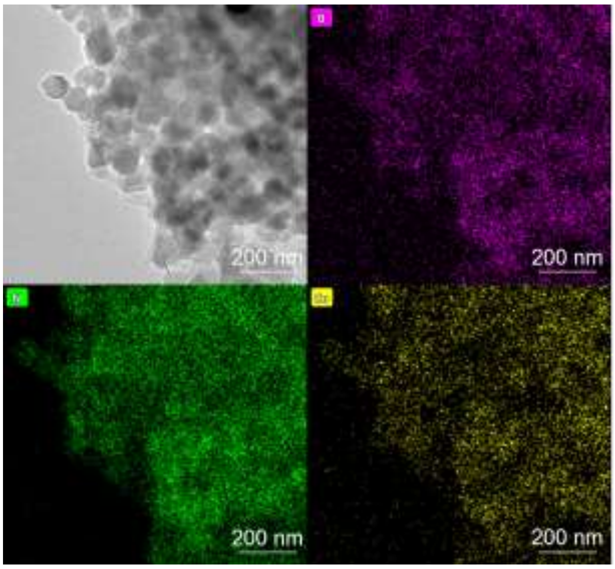

Figure S5 The TEM of ZIF-67@BNNS.

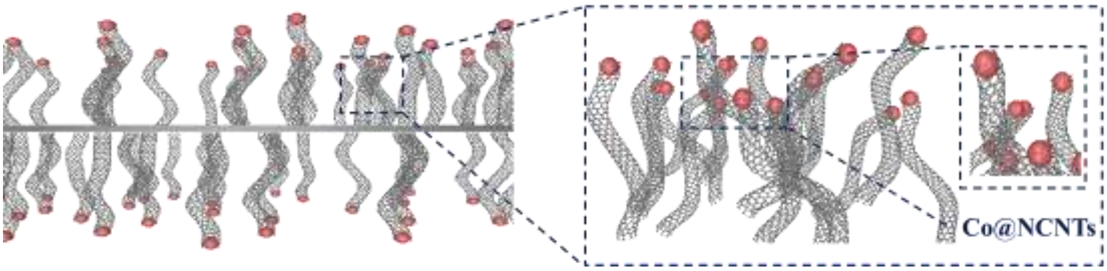

Figure S6 schematic illustration forCo@NCNTs

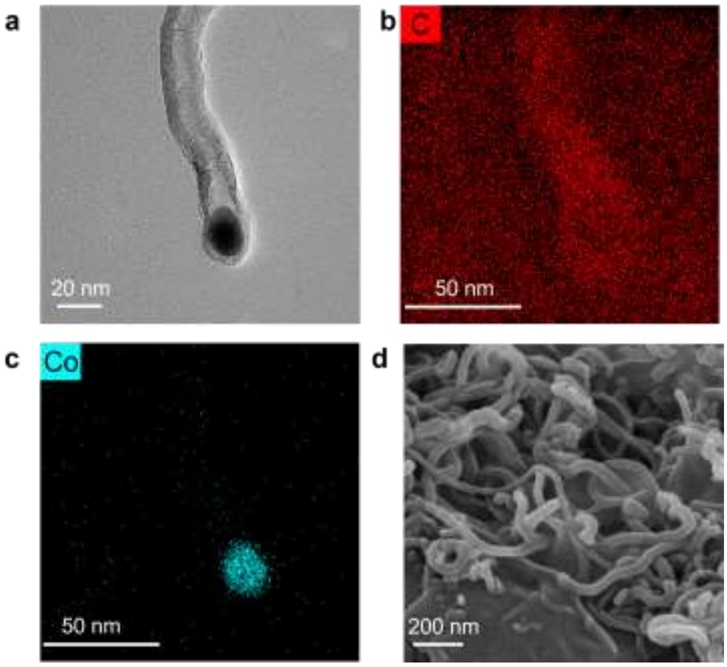

Figure S7 TEM (a), EDS(b-c) and SEM images of Co@NCNTs.

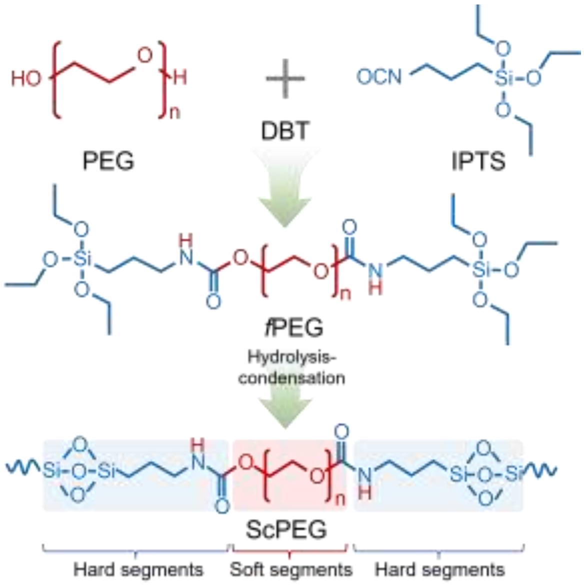

Figure S8 The synthetic route of ScPEG.

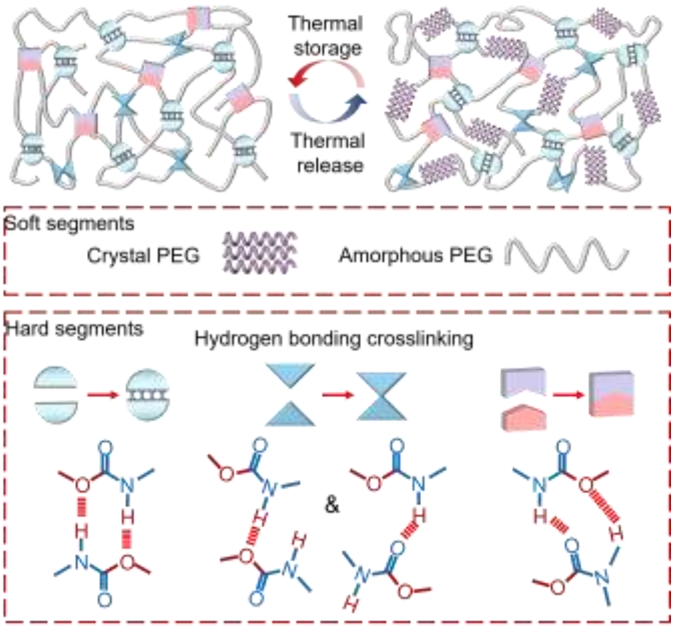

Figure S9 Schematic diagram of the phase change process and multiple hydrogen bonds crosslinking.

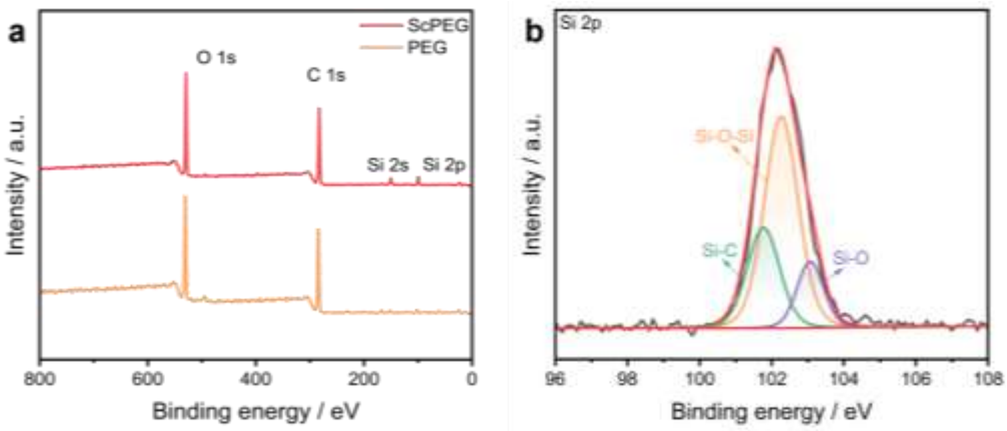

Figure S10 XPS full spectra (a) of PEG and ScPEG; Si 2p spectra (b) of ScPEG.

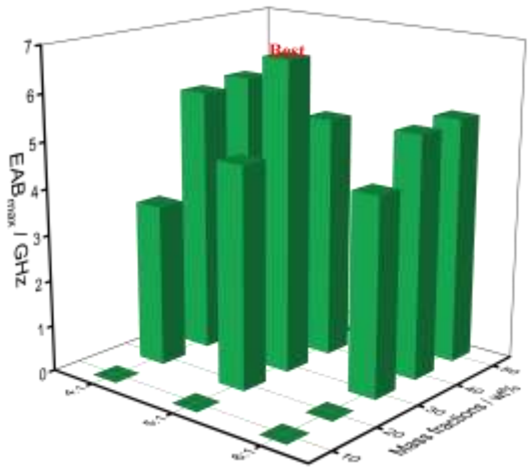

Figure S11 Comparison of EAB_{max} with different ratios of BNNS and Co^{2+} at different mass fractions.

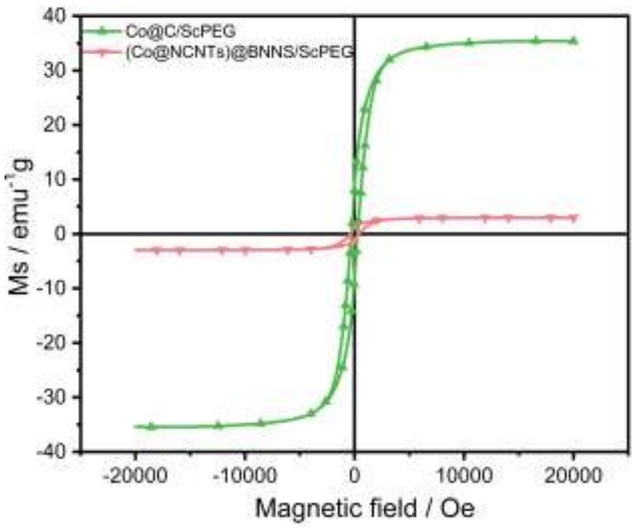

Figure S12 Hysteresis loops of Co@C/ScPEG and (Co@NCNTs)@BNNS/ScPEG composites.

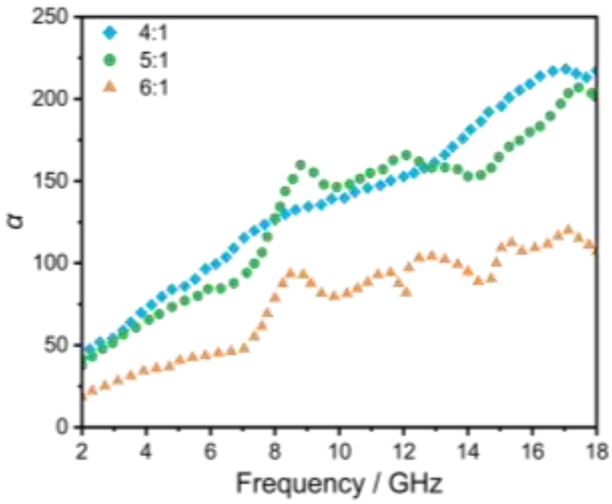

Figure S13 α of (Co@NCNTs)@BNNS/ScPEG composites prepared when the mass ratios of BNNS to Co^{2+} are 4:1, 5:1 and 6:1.

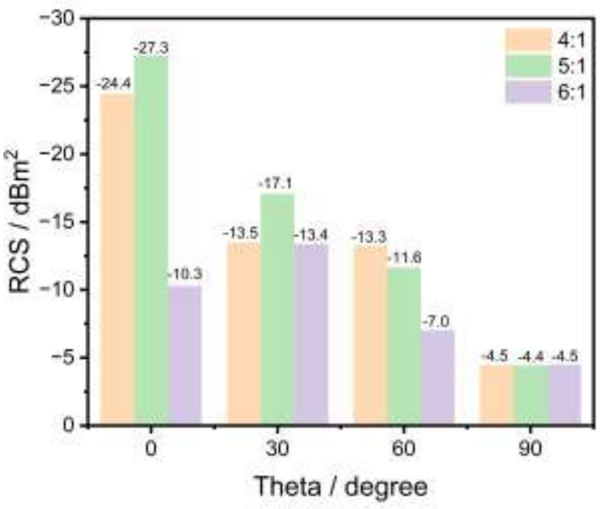

Figure S14 RCS reduction values of the (Co@NCNTs)@BNNS/ScPEG prepared when the mass ratios of BNNS to Co^{2+} are 4:1, 5:1 and 6:1.

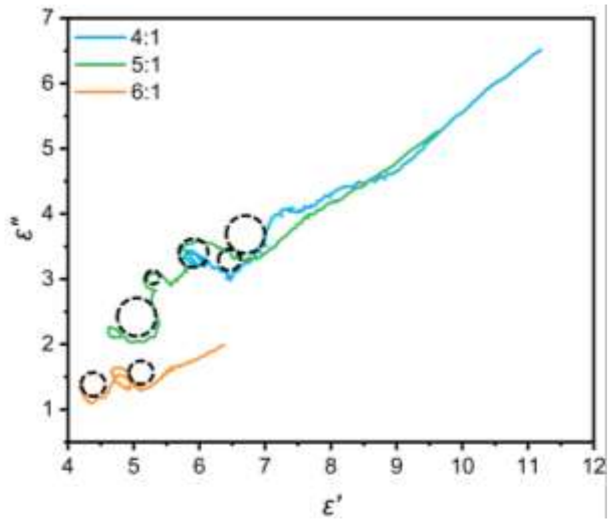

Figure S15 ε'-ε'' curves of (Co@NCNTs)@BNNS/ScPEG composites prepared when the mass ratios of BNNS to Co^{2+} are 4:1, 5:1 and 6:1.

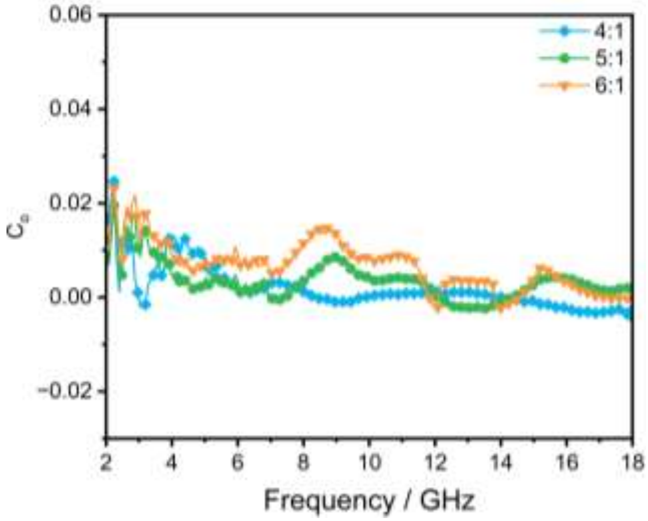

Figure S16 C_0 values of (Co@NCNTs)@BNNS/ScPEG prepared when the mass ratios of BNNS to Co^{2+} are 4:1, 5:1 and 6:1.

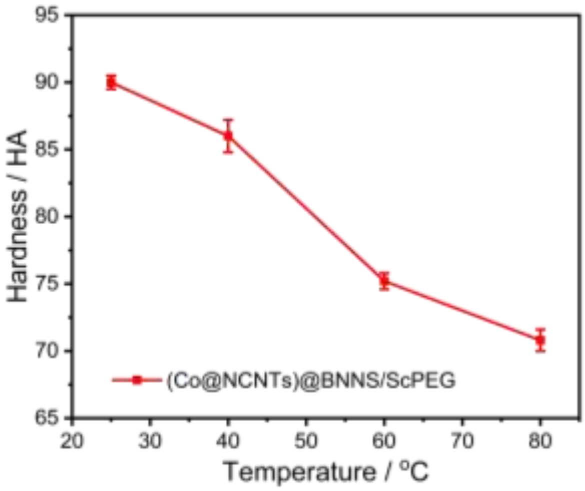

Figure S17 The hardness of (Co@NCNTs)@BNNS/ScPEG composites versus heating time.

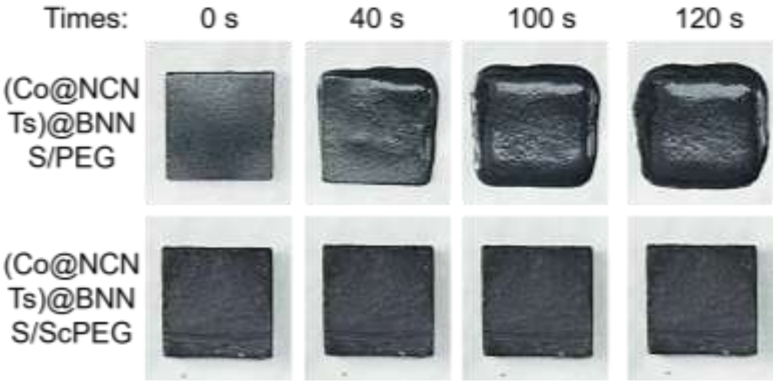

Figure S18 Photographs of (Co@NCNTs)@BNNS/ScPEG composites and (Co@NCNTs)@BNNS/PEG composites at different temperature.

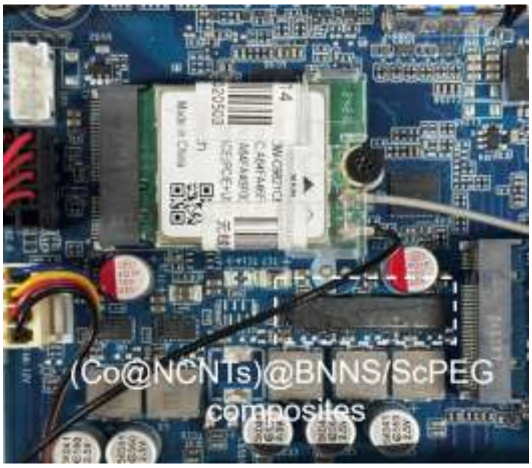

Figure S19 Schematic diagram of the structure in the overclocking computer motherboard.

Table S1 Comparison of λ of different materials.

Type	Materials	Mass fracctions (wt%)	λ W/(m K)	Ref.
Ceramic fillers	(SiC-BNNS)/epoxy	20	0.89	[1]
	BNNS@SiCnws/epoxy	20	1.17	[2]
	AlN@BN/epoxy	60	1.45	[3]
	GBN/epoxy	5	0.48	[4]
	ND@SiC-PVDF	70<br>60<br>50<br>40<br>30	2.39<br>1.98<br>1.05<br>0.62<br>0.48	[5]
	ND@SCNWs/PDMS	25<br>20	0.57<br>0.45	[6]
	s-BN/PDMS	35<br>30<br>20	1.16<br>0.74<br>0.59	[7]
	M-BN/C/epoxy	23<br>20<br>18<br>12<br>8	1.52<br>1.14<br>0.94<br>0.62<br>0.45	[8]
	BN-PVDF/epoxy	21<br>16<br>12<br>10<br>5	1.45<br>1.12<br>0.75<br>0.54<br>0.38	[9]
	POSS-G-SiCp/UHMWPE	20<br>30<br>40<br>50<br>60	0.68<br>0.75<br>1.14<br>1.32<br>1.52	[10]
Carbon fillers	EG/CNTs	18	2.06	[11]
	RGO/Fe_3O_4	8.97	1.21	[12]
	BNNS/CNTs	50<br>30<br>15	2.78<br>1.05<br>0.35	[13]
	GNPs/RGO/epoxy	20.4<br>15.4<br>8	1.56<br>1.28<br>0.75	[14]
Carbon-metal fillers	APCF-epoxy	5	2.05	[15]
	**(Co@NCNTs)@BNNS /ScPEG**	**30**	**2.55**	***This work***

Table S2 Comparison of microwave absorption performance of different materials.

Type	Materials	EAB_{max} / GHz	λ W/(m K)	Ref.
**Single**	CIP/Al_2O_3/NBR	3.54	1.66	[16]
	CIP/Al_2O_3/SR	3	2	[17]
	Al_2O_3/ZnO/SR	4	1.5	[18]
**heterostructure**	CNT@NiO/NR	4.24	1.03	[19]
	BCN/NR	4.16	0.28	[20]
	TiO_2@C-Ni/CNTs/NR	5.5	0.25	[21]
	BN@C/Ag	6.89	0.42	[22]
	HO-BNNS@Fe_3O_4	2.5	1.75	[23]
	BN-o/rGO@Fe_3O_4@PAn	3.12	1.69	[24]
	BN/Ni/CNT/WPU films	3.12	0.76	[25]
	BN@NC/WPU films	2.88	0.92	[26]
**3D network heterostructure**	CNF@C-Ni/EP	5.68	0.5	[27]
	CA/m-SiC/m-BN/EP	2.8	2.21	[28]
	MDCF@hBN/EP	5.6	0.99	[29]
	**(Co@NCNTs)@BNNS/ScPEG**	**6.72**	**2.51**	***This work***

Experimental Section/Methods

1 Main material

Cobaltous nitrate hexahydrate ($Co(NO_3)_2$ $6H_2O$) and 2-methylimidazole (2-MI) were all obtained from Macklin Biochemical Technology Co., Ltd. (Shanghai, China). Methanol was purchased from Jinhuada Chemical Reagent Co., Ltd. (Guangdong, China). Boron nitride (h-BN, diameter approximately of 20 μm) was purchased from Shanghai Xiangtian Nanomaterials Co., Ltd. (Shanghai, China). 4A molecular sieves were supplied by Sinopharm Chemical Reagent Co., Ltd. Polyethylene glycol (PEG, M_n=10000), dibutyltin dilaurate (DBT, 95%), and 3-Isocyanatopropyltriethoxysilane (IPTS, 95%) were provided by Aladdin Chemical Reagent Co., Ltd. (Shanghai, China). All chemicals were analytical grade and used without further purification.

2 Preparation of ZIF-67@BNNS and (Co@NCNTs)@BNNS

boron nitride (h-BN) was mixed with urea at a mass ratio of 1:30 and ball milled at 500 rpm for 24 hrs at room temperature. The resulting powder was dispersed in deionized water, washed several times by centrifugation to remove unreacted urea, and subsequently freeze-dried to obtain BNNS-NH_2 . Next, 2-MI (1.056 g) was weighed and dissolved in CH_3OH (30 mL) to conFig. Solution A. Solution B was conFig.d by weighing $Co(NO_3)_2$ $6H_2O$ (0.21 g) and a certain amount of BNNS-NH_2 dissolved in CH_3OH (20 mL). The mass ratios of BNNS to Co^{2+} were 4:1, 5:1 and 6:1, respectively. Solution A and solution B were well mixed and stirred for 4 hrs to obtain ZIF-67@BNNS. Finally, ZIF-67@BNNS and melamine were mixed at a mass ratio of 1:3 and pyrolyzed at 700°C for 2 hrs (2 ^{o}C/min, Ar/H_2) to obtain (Co@NCNTs)@BNNS heterostructural fillers.

3 Preparation of ScPEG

First, the original PEG and toluene solvents were dehydrated. Specifically, PEG was dried in an oven for 24 hrs, and toluene was dehydrated using a 4A molecular sieve. Then,

PEG was slowly dissolved in toluene at 70^oC (100 mg/mL). Subsequently, IPTS (159 μL) and DBT (75 μL) were slowly dropped into the above solution (40 mL) and stirred at 40^oC for 6 hrs. Toluene was removed by rotary evaporation to obtain PEG with active end groups. Finally, PEG with active end groups was dissolved in hydrochloric acid solution (0.1 mol/L) and stirred at 60^oC for 3 hrs to obtain ScPEG solution.

4 Preparation of (Co@NCNTs)@BNNS/ScPEG composites

The suspension, formed by mixing (Co@NCNTs)@BNNS heterostructural fillers with ScPEG solution, was poured into a custom-made mold equipped with a silicone rubber wedge and subsequently frozen. After the suspension was completely frozen, (Co@NCNTs)@BNNS/ScPEG-oriented framework was prepared via freeze-drying. Finally, the oriented framework was hot-pressed to obtain (Co@NCNTs)@BNNS/ScPEG composites.

5 Preparation of (Co@NCNTs)@BNNS/PEG composites

Heat the PEG powder at 80^oC until completely melted into a liquid state. Then, (Co@NCNTs)@BNNS heterostructure filler was thoroughly mixed with PEG liquid and cooled to room temperature to obtain (Co@NCNTs)@BNNS/PEG powder. The powder was placed in a custom-made mold and pressed (2 Mpa) to obtain (Co@NCNTs)@BNNS/PEG composites.

6 Preparation of r-(Co@NCNTs)@BNNS/ScPEG and BNNS/ScPEG composites

The (Co@NCNTs)@BNNS heterostructure fillers were thoroughly mixed with ScPEG solution and placed in a vacuum drying oven at 70^oC, and the randomly dispersed composites (r-(Co@NCNTs)@BNNS/ScPEG) were obtained after the solvent volatilized.

7 Preparation of BNNS/ScPEG composites

The mixed suspension with BNNS and ScPEG is poured into a custom-made mold with a silicone rubber wedge and frozen (-196^oC). After the suspension is completely frozen, the

BNNS/ScPEG oriented framework is prepared by lyophilization. Subsequently, the BNNS/ScPEG composites were obtained after hot pressing.

8. Characterizations

Fourier transform infrared (FT-IR) spectra of the samples were obtained on Bruker Tensor 27 equipment (Bruker Corp., Germany). The characterization method was attenuated total internal reflectance (ATR) with the testing range of 500-4000 cm^{-1}. X-ray diffraction (XRD) patterns of the samples were tested using an XRD (D8 Advance, Bruker, Germany) machine equipped with a Cu-targeted radiation source , scanning speed was 10 ^{o}/min with the diffraction angle of 2θ = 5° to 80°. The step size was 0.02 ^{o}/step. Raman spectra of the samples were performed on Alpha300R type confocal Raman spectrometer (WITec Co., Germany). X-ray photoelectron spectroscopy (XPS) analyses of the samples were investigated on AXIS Ultra DLD type device (Kratos Co., UK). Scanning electron microscopy (SEM) images of the samples were captured on Gemini 300 type equipment (Zeiss Co., Germany). Transmission electron microscopy (TEM) images of the samples were collected on Talos F200X type TEM (FEI Co., USA). The enthalpy change of the sample the samples were characterized using a differential scanning calorimetry (DSC, DSC3, Mettler-Toledo, Switzerland), heating rate was 10 ^{o}C/min and the atmosphere was nitrogen.

In-plane thermal conductivity coefficient ($\lambda_{//}$) and through plane thermal conductivity coefficient ($\lambda_{\perp}$) were measured using TPS2200 Hot Disk thermal constant analyzer (AB Corp., Sweden), according to standard ISO 22007-2:2008. The sensor type, working power and characteristic time for characterizing $\lambda_{//}$ 和 $\lambda_{\perp}$ were No. 7577, 10 mW and 5 s. The sample size is 20 mm×20 mm×2.5 mm. The surface temperature of the samples was recorded by Ti 300 infrared thermal imager (Fluke Co., USA). Magnetic hysteresis loops of the samples were obtained by vibrating sample magnetometer (LakeShore Corp., America) with magnetic field strength of -20000~20000 Oe. Electromagnetic parameters of the samples were measured

using MS46322B vector network analyzer (Anritsu Co., Japan), which used the coaxial method by a circular cylinder with an inner diameter of 3 mm and an outer diameter of 7 mm according to ASTM D7449. Herein, The (Co@NCNTs)@BNNS fillers were dispersed in ScPEG matrix with a mass fraction of 30 wt%.

The permittivity and permeability were measured by the coaxial method based on the transmission line theory. The reflection loss (RL) is calculated (**Equation (S1)**) at different thicknesses in the range of 2 to 18 GHz.

$$\mathrm{RL}=20\log\left|\frac{(Z_{in}-Z_0)}{(Z_{in}+Z_0)}\right| \qquad \textbf{Equation (S1)}$$

$$Z_{in}=Z_0\sqrt{\frac{\mu_r}{\varepsilon_r}}\tanh\left[j\left(\frac{2\pi f d}{c}\right)\sqrt{\mu_r\varepsilon_r}\right] \qquad \textbf{Equation (S2)}$$

Z_0 and Z_{in} (**Equation (S2)**) are the characteristic impedance of free space and the normalized input impedance of the sample, *f* and c are the microwave frequency and the speed of light in free space, ε_r is the relative complex permittivity (ε_r=ε'-ε''), μ_r is the relative complex permeability (μ_r=μ'-μ'') and d is the sample thickness. In actual applications, it is generally required that the microwave absorption materials can absorb 90% of the incident microwave, i.e., the RL value should be less than -10 dB.

The well impedance matching between the material and the space allow the microwave enter the interior of the material as much as possible and obtain the better microwave absorption efficiency. The impedance match degree (|Δ|, **Equation (S3)**) is calculated by the delta function method as follows where K and M are calculated from the electromagnetic parameters. Impedance matching is indicated the balanced impedance for |Δ| less than 0.4.

$$|\Delta|=|\sinh^2(Kfd)-M| \qquad \textbf{Equation (S3)}$$

The magnitude of α (**Equation (S4)**) reflects the materials reflection loss capability to microwave. Under the alternating magnetic field, currents are often induced in the material,

causing eddy current losses (C_0, **Equation (S5)**). If the magnetic loss of the sample to microwave is caused only by eddy current losses, then the C_0 does not vary with frequency.

$$\alpha=\sqrt{2}\pi f/c\sqrt{(\mu''\varepsilon''-\mu'\varepsilon')+\sqrt{(\mu''\varepsilon''-\mu'\varepsilon')^2+(\mu'\varepsilon''+\mu''\varepsilon')^2}} \qquad \textbf{Equation (S4)}$$

$$C_0=\mu''/(f\mu'^2)=2\pi\mu_0\sigma d^2/3 \qquad \textbf{Equation (S5)}$$

9 Charge differential density simulation based on DFT of (Co@NCNTs)@BNNS

The density functional theory (DFT) calculations were performed using the Vienna ab initio simulation package (VASP). The interaction between valence electrons and ionic cores was described using the projector augmented wave (PAW) method. The Perdew-Burke-Ernzerhof (PBE) exchange-correlation functional within the generalized gradient approximation (GGA) was employed to account for the electron-electron interactions. A kinetic energy cutoff of 400 eV with spin-polarization was used to expand the wave function of the valence electronic states. The convergence criteria for the electronic steps were set to 1×10^{-6} eV/atom, and for the ionic steps, the maximum force was set to be less than 0.05 eV/Å. The Brillouin zone was sampled using Monkhorst-Pack k-point meshes of $4\times4\times1$. Dipole corrections were included in all the calculations to minimize the inaccuracies in the total energy due to the simulated slab interactions. The dipole moment was calculated parallel to the z-direction. The empirical correction in Grimme's method with Becke-Jonson damping (DFT-D3(BJ)) was used to describe the van der Waals interactions. BN layer on Co (1 1 1), the calculation supercell contained 4 layers of Co (1 1 1) substrate lattices and 2 layer BN as Fig. 6e.

References

1. Han YX, Shi XT, Yang XT *et al.* Enhanced thermal conductivities of epoxy nanocomposites via incorporating in-situ fabricated hetero-structured SiC-BNNS fillers. *Compos Sci Technol* 2020; **187**: 107944.
2. Han YX, Shi XT, Wang SS *et al.* Nest-like hetero-structured BNNS@SiCnws fillers and significant improvement on thermal conductivities of epoxy composites. *Composites Part B* 2021; **210**: 108666.
3. Yao T, Chen K, Shao T *et al.* Nano-BN encapsulated micro-AlN as fillers for epoxy composites with high thermal conductivity and sufficient dielectric breakdown strength. *IEEE Trans Dielectr Electr Insul* 2020; **27**: 528-34.
4. Ren JW, Li QH, Yan L *et al.* Enhanced thermal conductivity of epoxy composites by introducing graphene@boron nitride nanosheets hybrid nanoparticles. *Mater Des* 2020; **191**: 108663.
5. Guan CL, Qin Y, Bo W *et al.* Highly thermally conductive polymer composites with barnacle-like nano-crystalline Diamond@Silicon carbide hybrid architecture. *Composites Part B* 2020; **198**: 108167.
6. Qin Y, Wang B, Hou X *et al.* Constructing tanghulu-like diamond@silicon carbide nanowires for enhanced thermal conductivity of polymer composite. *Compos Commun* 2022; **29**: 101008.
7. Li M, Wang MJ, Hou X *et al.* Highly thermal conductive and electrical insulating polymer composites with boron nitride. *Composites Part B* 2020; **184**: 107746.
8. Pan D, Li QM, Zhang W *et al.* Highly thermal conductive epoxy nanocomposites filled with 3D BN/C spatial network prepared by salt template assisted method. *Composites Part B* 2021; **209**: 108609.
9. Chen XL, Lim JS, Yan WL *et al.* Salt template assisted BN scaffold fabrication toward highly thermally conductive epoxy composites. *ACS Appl Mater Interfaces* 2020; **12**: 16987-96.
10. Gu JW, Guo YQ, Lv ZY *et al.* Highly thermally conductive POSS-g-SiCp/UHMWPE composites with excellent dielectric properties and thermal stabilities. *Composites Part A* 2015; **78**: 95-101.
11. Guo YL, Zhang RZ, Wu K *et al.* Preparation of nylon MXD_6/EG/CNTs ternary composites with excellent thermal conductivity and electromagnetic interference shielding effectiveness. *Chin J Polym Sci* 2017; **35**: 1497-507.

12. Liu YC, Lu MP, Wu K *et al.* Anisotropic thermal conductivity and electromagnetic interference shielding of epoxy nanocomposites based on magnetic driving reduced graphene oxide@Fe_3O_4. *Compos Sci Technol* 2019; **174**: 1-10.
13. Tian XJ, Pan T, Deng BJ *et al.* Synthesis of sandwich-like nanostructure fillers and their use in different types of thermal composites. *ACS Appl Mater Interfaces* 2019; **11**: 40694-703.
14. Liang CB, Qiu H, Han YY *et al.* Superior electromagnetic interference shielding 3D graphene nanoplatelets/reduced graphene oxide foam/epoxy nanocomposites with high thermal conductivity. *J Mater Chem* 2019; **7**: 2725-33.
15. Li JP, Qi SH, Zhang MY *et al.* Thermal conductivity and electromagnetic shielding effectiveness of composites based on Ag-plating carbon fiber and epoxy. *J Appl Polym Sci* 2015; **132**: 42306.
16. Wang XW, Shi ZF, Li YR *et al.* Analysis of absorption and thermal conductivity characteristics of composite patches based on Al_2O_3/carbonyl iron powder. *Magnetic Mater devices* 2021; **52**: 24-29.
17. Zou HZ, Wan WT, Yang MH *et al*. Composite powder preparation and characterization of thermal conductive wave-absorbing materials. *Polymer Mater* 2019; **35**: 136-40.
18. Zheng K, Jia K, Liu W *et al*. Research on the functional characteristics and application of thermal conductive wave-absorbing silicone rubber. *New Chem Mater* 2020; **48**: 77-80+84.
19. Zhou MF, Wan GP, Mou PP *et al.* CNT@NiO/natural rubber with excellent impedance matching and low interfacial thermal resistance toward flexible and heat-conducting microwave absorption applications. *J Mater Chem* 2021; **9**: 869-80.
20. Mou PP, Zhao JC, Wang GZ *et al.* BCN nanosheets derived from coconut shells with outstanding microwave absorption and thermal conductive properties. *Chem Eng J* 2022; **437**: 135285.
21. Wu LH, Liu X, Wan GP *et al.* Ni/CNTs and carbon coating engineering to synergistically optimize the interfacial behaviors of TiO_2 for thermal conductive microwave absorbers. *Chem Eng J* 2022; **448**: 137600.
22. Luo C, Fei DZ, Zhou JT *et al.* Silver particle-modified BN@C composites for thermal management and microwave absorption. *Ceram Int* 2024; **50**: 22252-65.
23. Bai YF, Liu R, Wang LX *et al.* Microwave absorption and thermal conductivity properties of HO-BNNS@Fe_3O_4 composites. *J Alloys Compd* 2020; **837**: 155574.

24. Bai YF, He LL, Lv P *et al.* Impedance-matched (hydroxylated nano-BN/reduced graphene oxide) Fe_3O_4/polyaniline composite for efficient microwave absorption and thermal management. *Mater Chem Phys* 2023; **295**: 127193.
25. Li ZX, Yang W, Chen Z *et al.* Boron nitride and carbon nanotube-bridged interfaces for boosting thermal conduction and electromagnetic wave absorption. *ACS Appl Nano Mater* 2024; **7**: 4264-76.
26. Li ZX, Yang W, Jiang B *et al.* Engineering of the core-shell boron nitride@nitrogen-doped carbon Heterogeneous interface for efficient heat dissipation and electromagnetic wave absorption. *ACS Appl Mater Interfaces* 2023; **15**: 7578-91.
27. Qian YX, Tao Y, Li W *et al.* High electromagnetic wave absorption and thermal management performance in 3D CNF@C-Ni/epoxy resin composites. *Chem Eng J* 2021; **425**: 131608.
28. Pan D, Yang G, Abo-Dief HM *et al.* Vertically aligned silicon carbide nanowires/boron nitride cellulose aerogel networks enhanced thermal conductivity and electromagnetic absorbing of epoxy composites. *Nano-Micro Lett* 2022; **14**: 118.
29. Qian YX, Tao Y, Li Y *et al.* High performance epoxy resin with efficient electromagnetic wave absorption and heat dissipation properties for electron packaging by modification of 3D MDCF@hBN. *Chem Eng J* 2022; **441**: 136033.
